# Evaluation of cognitive function in patients with severe anorexia nervosa before and after medical stabilization

**DOI:** 10.1186/s40337-020-00312-5

**Published:** 2020-07-31

**Authors:** Melanie Rylander, Gillian Taylor, Susan Bennett, Christopher Pierce, Angela Keniston, Philip S. Mehler

**Affiliations:** 1 Departments of Internal Medicine and Psychiatry, Denver Health Medical Center, University of Colorado School of Medicine, 777 Bannock St, Denver, CO 80204 USA; 2 Department of Psychiatry, Denver Health Medical Center, University of Colorado School of Medicine, Denver, CO 80204 USA; 3 Department of Internal Medicine, University of Colorado School of Medicine, Denver, CO 80204 USA; 4 ACUTE Center for Eating Disorders, Denver Health Medical Center, University of Colorado School of Medicine, Denver, CO 80204 USA

**Keywords:** Anorexia nervosa, Malnutrition, Cognition, Refeeding, Starved brain

## Abstract

**Background:**

The purpose of this study was to quantify cognitive deficits in severe anorexia nervosa (AN) before and after medical stabilization.

**Methods:**

This was a prospective study of 40 females between the ages of 18 and 50 admitted to a medical stabilization unit with severe AN (%IBW < 70). The primary outcome of the study was change in test scores on the Repeatable Battery for the Assessment of Neuropsychological Status (RBANS) at baseline and after medical stabilization.

**Results:**

There were no statistically significant differences in baseline RBANS scores between AN patients overall and controls (*p* = 0.0940). There was a statistically significant change in RBANS from baseline 94.1 + 12.7 to medical stabilization 97.1 + 10.6 (*p* = 0.0173), although notably both mean values fell within the average range. There were no significant differences in baseline RBANS scores between controls and AN-BP patients (*p* = 0.3320) but significant differences were found between controls and AN-R patients (*p* = 0.0434).

**Conclusions:**

No baseline deficits in cognition were found in this sample of women with severe AN.

## Plain English summary

This study sought to identify impairments in cognition in women suffering from severe anorexia nervosa (AN), binge purge subtypes (AN-BP) and restricting subtype (AN-R), relative to healthy volunteers. All subjects were patients on a medical refeeding unit with severe illness. Cognitive testing occurred upon entry into the program and at exit. Surprisingly, our study did not find any difference in baseline cognition between patients with AN as a whole and health volunteers. Patients with AN-R had significant improvements in cognition, but this is likely not clinically relevant.

## Background

Anorexia nervosa (AN) is associated with adverse effects on cognitive functioning in the domains of attention, processing speed, visual and verbal memory, and visuospatial construction [1–5], as well as high rates of comorbid anxiety, depression, and obsessive compulsive disorder [1, 6, 7]. Both mental and physical stabilization are essential for full recovery from AN [8–10]. Several neuropsychological studies that have reported cognitive impairment in individuals with anorexia nervosa before formal treatment subsequently noted improvement in many facets of cognitive functioning following weight restoration [2–5]. However, the findings noted above have been discordant from those of other studies. Two studies found no change in cognitive functioning with weight restoration [11, 12]. Another study found differences in baseline cognition between patients with AN and healthy controls, but no improvement in cognition with weight restoration [13]. Additionally, investigators have found that impairments in the cognitive domain of memory performance persist after weight restoration [14]. The inconsistencies in findings may be due to different patient populations, study designsvarying assessment tools, and patients being studied at different stages of weight recovery. No studies have examined the cognitive function of a patient population with severe AN defined as an ideal body weight less than 70% (%IBW < 70) before and after medical stabilization. The degree to which cognitive function is variably compromised in patients with severe forms of AN remains unknown.

Only two other studies have quantified the difference in change of cognitive function between patients with the two distinct subtypes of AN, the restricting subtype and the binge-purge subtype (R and BP) [3, 14]. These studies report contrasting results. Since medical treatments, lengths of stay, and course of recovery differ for the two groups, compromised cognitive functions in the two populations are likely to differ. No prior studies have examined differences between AN-R and AN-BP in highly medically compromised populations of patients with AN.

The prognosis of AN in adults is poor, with less than half of patients reaching recovery [15]. Standardized mortality ratios for patients with AN range from 4.4–14 [8, 16–18], suggesting that current therapies for AN do not facilitate recovery in the majority of patients. Potentially contributing to poor outcomes, severely ill patients may not be able to fully participate in current standard of care psychotherapies because they are largely cognitive based.

Leptin is an adipokine traditionally thought to be involved in energy regulation [19]. Several studies have found leptin deficiencies in patients with AN and subsequent increases in leptin with refeeding [20–24]. Recently, animal studies have demonstrated that leptin has a role in synaptogensis and neurogenesis in the hippocampus [25–28]. Furthermore, deficits in learning and memory in leptin deficient mice have been corrected with leptin administration [25]. Leptin’s observed role in neurogenesis and learning raises questions about whether leptin deficiency is a mediator of cognitive deficits observed in AN.

The primary aims of this study were four-fold:

1. Quantify baseline cognitive deficits in patients with severe AN in the domains of immediate memory, delayed memory visuospatial/constructional, language, and attention. We hypothesized that patients with severe AN would have deficits in these domains relative to healthy controls.
2. Evaluate changes in immediate memory, delayed memory visuospatial/constructional, language, and attention from baseline through the end of acute refeeding and medical stabilization in patients with severe AN. We hypothesized that after medical stabilization performance in the cognitive domains tested in patients with AN would significantly improve.
3. Evaluate and compare baseline and change in immediate memory, delayed memory visuospatial/constructional, language, and attention by AN subtype (binge-purge (BP) vs. restricting (R) after medical stabilization. We hypothesized that cognitive rebound after medical stabilization would be greater in the BP subtype compared to the R subtype in the domains tested.
4. Evaluate correlations between changes in immediate memory, delayed memory, visuospatial/constructional, language, and attention from baseline to medical stabilization with changes in serum leptin levels from baseline to medical stabilization after controlling for changes in weight. We hypothesized there would be a positive correlation between changes in cognitive domains and changes in serum leptin levels after controlling for changes in weight.

This is the first study to evaluate change in cognitive deficits in patients with severe AN, compare differences in cognitive function between AN subtypes, and evaluate leptin as a possible mediator of cognitive impairment in severe malnutrition.

## Materials and methods

### Patient population

This prospective, single-center, non-randomized design was conducted between July 2016 and October of 2018. All patients (*n* = 510) between the ages of 18 years and 50 years who presented to the ACUTE Center for Eating Disorders at Denver Health Medical Center were screened for inclusion. Inclusion criteria were chosen to minimize confounders of older age on cognitive function and barriers inherent to conducting cognitive testing in non-English speaking patients. Exclusion criteria were chosen to eliminate patients in whom cognitive testing would not be possible or the results of such testing would be rendered invalid because of comorbid factors known to negatively impact cognition.

Inclusion Criteria included English speaking women between the ages of 18 and 50, a DSM 5 diagnosis of AN-R or AN-BP, and an IBW < 70%. Exclusion criteria included inability to give informed consent, inability to follow study protocol, neurologic disorders including multiple sclerosis, seizure disorders, dementias or mild cognitive impairment, delirium, history of traumatic brain injury, use of anti-epileptic medications, use of lithium, documented or self reported history of intellectual disability, active alcohol/substance use disorder, or alcohol/substance intoxication or withdrawal.

Age and gender matched controls were recruited from electronic and written advertisements sent to university and hospital list serves and posted around campus to match study subjects. Controls were screened for substance use disorders, neurologic disorders, seizure disorders, intellectual disabilities, and use of medications that might impair performance on cognitive testing via interview with a study team member. Controls were screened for eating disorders using Module H of the SCID as well a weight history to rule out those with a history of extreme weights or BMIs.

### Study assessments

All patients admitted to ACUTE were given standard of care treatment. Standard of care on the ACUTE unit includes collecting the following information upon admission and throughout hospitalization:

- Demographics
- Medical/Medication History
- Physical Exam
- Standard of Care ACUTE blood work
- Body Height
- Body Weight
- Vital Signs (blood pressure, heart rate, respiratory rate, temperature)
- Concomitant Medications (monitored routinely as per care standard)

Diagnosis of anorexia nervosa was made upon admission using DSM 5 diagnostic criteria by a licensed psychologist who specializes in diagnosis and treatment of eating disorders as per standard work flow on ACUTE [29]. Restricting and binge-purge subtypes were diagnosed upon admission based on current self-reported purging behavior, collateral history from outside sources, physical exam, and routine laboratory data.

### Outcomes

The primary outcome of the study was change in test scores on the Repeatable Battery for the Assessment of Neuropsychological Status (RBANS) at baseline and after medical stabilization.

Secondary outcomes included:

- Change in Trail Making Test, Part B/D (TMT-B/D) scores from baseline to medical stabilization
- Wide Range Achievement Test, (W-RAT 4) Word Reading Subtest at baseline
- Change in Beck Depression Inventory (BDI) scores from baseline to medical stabilization
- Change in Beck Anxiety Inventory (BAI) scores from baseline to medical stabilization
- Change in the Obsessive-Compulsive Inventory - Revised (OCI-R) scores from baseline to medical stabilization
- Changes in serum leptin levels and RBANS scores from baseline to medical stabilization after controlling for changes in weight.

### Standardized measures

Cognitive function:

- The RBANS [30] is a brief cognitive battery comprised of 12 subtests measuring 5 cognitive domains: immediate memory, delayed memory visuospatial/constructional, language, and attention. Four alternate forms of the RBANS are available, assessing the same cognitive domains with slightly different content. Two versions (Forms A and B) were administered in a counterbalanced fashion to control for order effects when doing longitudinal assessments. For half the patients RBANS A was administered within 3 days of admission to the ACUTE unit and RBANS B was administered once medical stabilization was achieved; the other half were first assessed with RBANS B followed by A.
- TMT-B [31] is a neuropsychological test of visual attention and task switching. TMT-D is an alternative form of TMT-B that can be used to minimize practice effect when doing longitudinal assessments [32].
- WRAT-4 [33] Word Reading Subtest was used to measure cognitive reserve, which is a predication of cognitive capacity before injury or illness, to control for baseline intelligence.
- The BDI [34], BAI [35], and OCI-R [36] were used to control for comorbid psychiatric symptoms that can impact cognition.

Medical stabilization: defined based on ACUTE protocol:

- The patient is consuming 2000–3000 kcal/day.
- Laboratory values indicate that end organ failure is resolved or resolving, and refeeding syndrome is no longer a concern.
- The patient has minimal edema and is physically strong enough to progress to eating disorder treatment programs.
- The patient is deemed medically stable for discharge by the treating physician.

### Sample size

The study required 20 patients in the AN BP group, 20 patients in the AN R group, and 10 controls for a total of 50 participants to test all study hypotheses and detect the pre-stated differences in outcomes. All power calculations assumed a type I error rate of 0.05, standard deviation of 9.4 for baseline RBANS test scores, standard deviation of 15.3 for post medical stabilization RBANS tests scores and a pooled standard deviation of 11.0 for the change in RBANS tests scores. A clinically meaningful difference in RBANS between independent groups was set at 13.1 [3]. A clinically meaningful difference in change in RBANS was set at 5.3 [5].

Sample sizes for testable hypotheses in the research aims were calculated such that the hypothesis test attained 95% power. Depending on the hypothesis, an independent samples t-tests or paired t-tests was used as the test statistic. The largest study sample size was taken as the sample size required to attain 95% power for all testable hypotheses.

### Test administration

Psychometric tests were administered within 3 days of admission by a licensed psychologist trained in neuropsychological assessments. Repeat psychometrics were performed by a licensed psychologist trained in neuropsychological assessments upon medical stabilization. All psychological testing was administered the same day as RBANS before and after medical stabilization. Alternate forms of the RBANS and TMT were administered in a counterbalanced manner at baseline and following medical stabilization to minimize any concern for administration order effects.

Quantitative serum leptin levels were obtained via chemiluminescent immunoassay performed by ARUP Laboratories. Leptin levels were obtained upon study entry and medical stabilization. Leptin levels were obtained the same day as psychometric tests were administered. Table 1 outlines the timeline of study specific events. Controls completed baseline psychometric testing and repeat psychometric testing within the same time frame as study patients. Controls did not undergo repeat leptin levels.

**Table 1:** Study Timeline (Testing occurs within 3 days of admission)

Baseline	Medical Stabilization
**Standard of Care Covariate information** • **Demographics** • **Medical/Medication History** • **Psychiatric History** • **Physical Exam** • **Body Height** • **Body Weight** • **BMI** • **Vital Signs** • **Complete metabolic panel and blood counts** • **Serum Prealbumin** • **Informed Consent**	**Standard of Care Covariate information** • **Medical/Medication History** • **Physical Exam** • **Body Height** • **Body Weight** • **BMI** • **Vital Signs** • **Complete metabolic panel and blood counts**
**Psychometric tests:** • **Wide Range Achievement Test 4, Word Reading Subtest** • **Repeatable Battery for the Assessment of Neuropsychological Status A or B** • **Beck Depression Inventory** • **Beck Anxiety Inventory** • **Obsessive Compulsive Inventory-Revised** • **Trail Making Test, Part B or D**	**Psychometric tests:** • **Repeatable Battery for the Assessment of Neuropsychological Status A or B** • **Beck Depression Inventory** • **Beck Anxiety Inventory** • **Obsessive Compulsive Inventory-Revised** • **Trail Making Test, Part B or D**
**Additional biomarker tests:** • **Serum Leptin**	**Additional biomarker tests:** • **Serum Leptin**
**AN-R = 20, AN-BP = 20, Controls = 10**	**AN-R = 20, AN-BP = 20,** **Controls = 10 (Psychometric tests only)**

### Statistical analysis

Patients were categorized by DSM 5 diagnosis of anorexia nervosa (AN) subtype, either binge-purge (AN-BP) or restricting (AN-R). Continuous variables were described using means and standard deviations (SD) or medians and interquartile ranges (IQR) based on tests of normality. Categorical variables were described using counts and frequencies.

Baseline data, specifically height, weight, BMI, and percent ideal body weight, were compared between cases and controls as well as between case subtypes using a Student’s t-test. W-RAT 4 for AN patients were compared to control participants using a Student’s t-test.

A paired t-test was used to test changes in the TMT-B/D score and Wilcoxon signed rank sum tests were used to evaluate changes in BDI-II total scores, BAI total scores, and OCI-R scores for checking, hoarding, neutralizing, obsessing, ordering, and washing from baseline to medical stabilization.

Linear regression modeling was used to assess the correlation between change in RBANS score and change in leptin values from baseline to after medical stabilization for AN patients, after controlling for changes in weight. Pearson Correlation Coefficients were used to assess correlations between improvements in RBANS score and improvements in the BDI-II total score, BAI total score, and OCI-R scores for checking, hoarding, neutralizing, obsessing, ordering, and washing for AN patients and for AN-BP and AN-R patients separately.

*P* values of < 0.05 were considered statistically significant, and all analyses were done using SAS Enterprise Guide software version 7.1 (SAS Institute, Cary, NC).

## Results

During the study period 510 patients were screened for eligibility. Fifty-seven patients met eligibility criteria and 39 patients completed the protocol. Four patients declined to participate in the study (one AN-BP patient and three AN-R patients), five patients were screen failures (four AN-BP patients and one AN-R patient), and nine patients withdrew from the study (five AN-BP patients and four AN-R patients). The average age of patients who did not participate in the study was 29 + 9 years of age.

Demographic characteristics of cases and controls are listed in Table 2, and anthropometric measurements are displayed in Table 3. There were no statistically significant differences in baseline weight, BMI or percent ideal body weight between AN-BP patients and AN-R patients. Baseline weights, BMI and percent ideal body weight differed significantly between AN-R and AN-BP patients and controls (*p* < 0.0001).

**Table 2:** Demographic Characteristics of Patients and Controls

	Patients			Controls
	***N*** = 39	AN-BP ***N*** = 19	AN-R ***N*** = 20	***N*** = 10
**Age, Mean** **+** **SD**	33 + 12	35 + 15	31 + 10	35 + 9
**Caucasian, N (%)**	39 (100)	19 (100)	20 (100)	9 (90)
**Hispanic, N (%)**	0 (0)	0 (0)	0 (0)	3 (30)
**Days Treatment** **+** **SD**		15 + 9	21 + 10	n/a

**Table 3:** Anthropometric Measurements of Patients and Controls

	Patients						Controls
	***N*** = 39		AN-BP ***N*** = 19		AN-R ***N*** = 20		***N*** = 10
	Pre	Post	Pre	Post	Pre	Post	Pre
**Height, Mean** **+** **SD**	64.7 + 2.7		64.2 + 2.6		65.0 + 2.7		64.2 + 2.7
**Weight (kgs), Mean** **+** **SD**	34.4 + 4.8	41.0 + 4.9	34.7 + 5.0	41.3 + 5.4	34.1 + 4.8	40.7 + 4.5	63.8 + 9.0
**BMI, Mean** **+** **SD**	12.7 + 1.1	14.6 + 1.2	12.9 + 1.2	14.8 + 1.2	12.5 + 1.1	14.4 + 1.3	24.0 + 3.6*
**% Ideal Body Weight**	61 + 5*	73 + 4*	62 + 5*	75 + 5*	60 + 5*	72 + 3*	117 + 20

*P < 0.0001difference relative to controls

### Aim 1: quantify baseline cognitive deficits in patients with severe AN

Results from neuropsychiatric assessments are presented in Table 4. W-RAT 4 scores at baseline for AN patients were compared to control participants using a Student’s t-test. No statistically significant differences were found (62.3 + 4.4 vs. 64.0 + 4.0, *p* = 0.2708) suggesting no differences in premorbid intelligence. There were no statistically significant difference in baseline RBANS scores between AN patients overall and controls (*p* = 0.0940) or in baseline TMT B/D scores between controls and AN patients overall (*p* = 0.125).

**Table 4:** Neuropsychiatric Assessments

	Patients						Controls	
	***N*** = 39		AN-BP ***N*** = 19		AN-R ***N*** = 20		***N*** = 10	
	Baseline	Post	Baseline	Post	Baseline	Post	Baseline	Post
**Wide Range Achievement Test (W-RAT 4) Word Reading Subtest, Mean** **+** **SD**	62.3 + 4.4		62.4 + 4.5		62.3 + 4.5		64.0 + 4.0	
**Repeatable Battery for the Assessment of Neuropsychological Status (RBANS) Total Score, Mean** **+** **SD**	94.1 + 12.6	97.1 + 10.6*	97.2 + 11.4	98.4 + 11.4	91.1 + 13.3**	95.8 + 10.0*	101.6 + 11.7	105.3 + 8.8
**Beck Depression Inventory (BDI-II) Total Score, Median (IQR)**	35 (30, 42)	20 (13, 28)*	33 (30, 39)	20 (15, 29)*	38 (29.5, 45)	19.5 (10, 26)*	3.5 (1, 5)	1 (0, 3)
**Beck Anxiety Inventory (BAI) Total Score, Median (IQR)**	25 (17, 33)	19 (12, 24)*	23 (14, 31)	19 (10, 21)*	29 (18.5, 40)	17.5 (14, 26.5)*	3 (1, 4)	1.5 (0, 4)
**Trail Making Test, Part B Score, Mean** **+** **SD**	34.7 + 10.5	39.5 + 10.5	37.4 + 8.6	40.8 + 10.1	32.2 + 11.7	38.2 + 10.9*	40.4 + 9.2	44.2 + 6.1
**Obsessive Compulsive Inventory – Revised (OCI-R), Median (IQR)**								
Checking	3 (2, 4)	2 (0, 3)*	3 (2, 6)	1 (0, 4) *	2 (1.5, 4)	2 (0.5, 2.5) *	0 (0, 0)	0 (0, 0)
Hoarding	4 (1, 6)	2 (0, 4)*	3 (0, 6)	2 (0, 6)	5 (1, 6)	2 (0, 2.5)*	1.5 (0, 3)	1 (0, 3)
Neutralizing	3 (1, 7)	1 (0, 6)*	1 (0, 6)	1 (0, 6)	5 (2, 7.5)	1.5 (0.5, 3.5)*	0 (0, 0)	0 (0, 0)
Obsessing	7 (3, 10)	3 (2, 7)*	6 (2, 10)	4 (1, 7)*	7 (3, 9.5)	3 (2, 7.5)*	1 (0, 3)	0 (0, 2)
Ordering	7 (5, 11)	6 (4, 9)	8 (5, 11)	7 (3, 9)	7 (4, 11)	6 (4, 11)	1.5 (0, 3)	2.5 (0, 3)
Washing	2 (0, 7)	1 (0, 4)*	2 (0, 7)	1 (0, 6)	1.5 (1, 7)	1.5 (0, 3.5)	0 (0, 0)	0 (0, 0)
**Leptin Level (ng/mL), N (%)**								
< 0.4	**31 (79)**	**19 (49)**	**16 (84)**	**10 (53)**	**15 (75)**	**9 (45)**		
0.4–1.0	7 (18)	10 (26)	3 (16)	6 (32)	4 (20)	7 (35)		
> 1.0	1 (3)	7 (18)	0 (0)	2 (11)	1 (5)	2 (10)		
< 6.0							4 (40)	
6.0–10.0							2 (20)	
> 11.0							3 (30)	
**Increase in Leptin Level, N (%)**	14 (36)		6 (32)		8 (40)			

*P < 0.05 change from baseline

**P < 0.05 relative to baseline of controls

### Aim 2: evaluate changes in cognitive functions from baseline through the end of acute refeeding and medical stabilization in patients with severe AN

For AN patients overall, there was a statistically significant change in RBANS from baseline (94.1 + 12.6) to medical stabilization (97.1 + 10.6) (*p* = 0.0173), although notably both mean values fell within the average range. When RBANS subscores were analyzed, only coding, story memory, and story recall showed statistically significant increases from baseline in AN patients as a whole (*p* < 0.004) (Table 5). No statistically significant change was found for control patients from baseline to post testing (*p* = 0.1955). No statistically significant change was found in TMT B/D scores from baseline through medical stabilization for AN patients as a whole.

**Table 5:** RBANS Subscores

	Cases						Controls	
	***N*** = 39		AN-BP ***N*** = 19		AN-R ***N*** = 20		Controls ***N*** = 10	
	Pre	Post	Pre	Post	Pre	Post	Pre	Post
**Repeatable Battery for the Assessment of Neuropsychological Status (RBANS), Mean** **+** **SD**								
List Learning Total Score	30 + 5	31 + 3	31 + 5	32 + 3	30 + 5	30 + 4	31 + 3	31 + 3
Story Memory Total Score	15 + 4	18 + 3*	16 + 4	19 + 3*	15 + 4	17 + 3	19 + 3	19.5 + 2
Figure Cope Total Score	19 + 2	19 + 1	19 + 1	19 + 1	19 + 2	19 + 2	19 + 1	19 + 1
Line Orientation Total Score	17 + 3	18 + 2	17 + 3	17 + 2	17 + 3	18 + 2	18 + 2	17.5 + 2
Picture Naming Total Score	10 + 1	10 + 1	9 + 1	10 + 1	10 + 1	10 + 1	10 + 0	10 + 1
Semantic Fluency Total Score	24 + 5	24 + 6	25 + 5	23 + 6	23 + 5	25 + 5	23 + 4	23 + 2
Digit Span Total Score	10 + 2	10 + 2	10 + 2	10 + 2	10 + 2	10 + 2	11 + 2	12 + 2
Coding Total Score	50 + 10	54 + 10*	51 + 8	55 + 9	49 + 13	54 + 11	55 + 10	61 + 9
List Recall Total Score	7 + 2	7 + 2	7 + 2	7 + 1	8 + 2	7 + 2	8 + 2	8 + 3
List Recognition Total Score	20 + 1	19 + 1	19 + 1	19 + 1	20 + 1	19 + 1	20 + 1	19.5 + 1
Story Recall Total Score	9 + 2	10 + 2*	10 + 2	10 + 2	8 + 2	10 + 2*	10 + 1	11 + 1
Figure Recall Total Score	15 + 4	16 + 3	16 + 2	17 + 2	15 + 4	15 + 3	15.5 + 4	17 + 2

*P < 0.004, adjusted for multiple comparisons

Statistically significant decreases in BDI and BAI scores were found for all AN patients (*p* < .0001). No statistically significant correlations were found between increases in RBANS score and changes in BDI or BAI scores for AN patients overall. Overall, AN patients showed significant decreases from baseline to medical stabilization in the OCI-R domains of checking (*p* < 0.05), obsessing (*p* < 0.05), hoarding (*p* < 0.02), neutralizing (*p* < 0.02), and washing (*p* < 0.02). No correlations between changes in RBANS score and OCI-R domains were found for AN patients overall. Controls did not show significant changes in OCI-R domains.

### Aim 3: evaluate and compare baseline and change in cognitive functions by AN subtype (binge-purge (BP) vs. restricting (R) after medical stabilization

There were no significant differences in baseline RBANS scores between controls (101.6 + 11.7) and AN-BP patients (97.2 + 11.4) (*p* = 0.3320) but significant differences were found between controls (101.6 + 11.7) and AN-R patients (91.1 + 13.3) (*p* = 0.0434). There were no significant differences between baseline TMT B/D scores in control versus AN-BP (*p* = 0.3881) or AN-R patients (*p* = 0.0628). In subgroup analysis, statistically significant changes in RBANS score from baseline to medical stabilization were found for AN-R patients (*p* = 0.0091) but not AN-BP patients (*p* = 0.4862). In addition, statistically significant changes in TMT B/D scores from baseline to medical stabilization were found for AN-R patients but not AN-BP patients (*p* = 0.0094 and *p* = 0.0995, respectively).

Statistically significant improvements in BDI and BAI scores were found for both AN-BP and AN-R patients (*p* < 0.001). No statistically significant correlations were found between improvements in RBANS score and changes in BDI or BAI scores for AN-BP or AN-R patients. For OCI-R domains, subgroup analysis showed significant changes in AN-R patients in the domains of checking (*p* < 0.05), obsessing (*p* < 0.05), hoarding (*p* < 0.001, and neutralizing (*p* < 0.0035). AN-BP patients showed significant reductions in checking (*p* < 0.05) and obsessing (*p* < 0.05). Across domains of the OCI-R (checking, hoarding, neutralizing, obsessing, ordering, and washing), we found a statistically significant correlation between improvements in RBANS and improvement in the washing domain (*p* = 0.0432) for AN-R patients only. No other correlations between changes in RBANS score and all other OCI-R domains were found in subgroup analysis.

### Aim 4: evaluate the correlation between changes in cognition from baseline to medical stabilization with changes in serum leptin levels from baseline to medical stabilization after controlling for changes in weight

Comparing AN patients and controls, there was no statistically significant difference in change in leptin from baseline to medical stabilization (*p* = 0.1573). Subgroup analysis showed similar findings. There was no difference in the proportion of AN-BP patients relative to control with change in leptin levels (*p* = 0.1266) or between controls and AN-R patients (*p* = 0.4197). After controlling for changes in weight, increases in RBANS score from baseline to after medical stabilization were not significantly associated with increases in leptin from baseline to medical stabilization for AN patients overall (*p* = 0.9661) or for either AN-BP patients (*p* = 0.9437) or AN-R (*p* = 0.9608) patients.

## Discussion

### General

This is the first study to examine cognitive deficits in patients with severe AN before and after medical stabilization. Our population is unique in the severity of malnutrition as evident in their admission %IBW as well as their stage of treatment at the time of enrollment. No prior studies have examined patients during acute refeeding on a medical inpatient setting.

Our study has several noteworthy findings. We found significantly lower admission cognitive scores in patients with severe AN-R relative to healthy controls and statistically significant increases in cognition after refeeding. The clinical significance of these differences is questionable. Normative data indicates RBANS Index scores of 90–109 are within the average range [37]. Both subgroups of patients and controls fell into this range at baseline and post testing. Although we hypothesized that cognitive deficits would interfere with the ability of patients in acute stages of illness to participate in cognitive based therapies, our data provides only limited support at best for this possibility. Analysis of subscores also did not show significant baseline deficits between patients and controls.

We found no differences in cognition between patients with AN-BP and healthy controls as measured by RBANS and TMT. Several previous studies demonstrating cognitive impairment in patients with AN have not investigated potential differences in diagnostic subtypes [2, 4, 11]. The lack of observed difference in baseline cognition between AN-BP and healthy controls was surprising given the significant differences in body weight. As we discuss below, body weight is likely not a complete proxy for nutritional status.

Though cognitive scores improved in our total patient cohort, subgroup analysis revealed that these improvements were only observed in patients with AN-R. The lack of significant improvement in AN-BP patients was not unexpected given the absence of significant baseline impairments. The improvements in AN-R patients remained significant after controlling for changes in BAI, BDI, and OCI-R scores suggesting improvement in comorbid psychiatric symptoms did not account for improvements in cognition. The noted improvement in AN-R patients cannot be attributed to poorer nutritional status at baseline as measured by body weight as we found no significant difference between patients with AN-R and AN-BP in admission BMI or %IBW.

These findings suggest that weight is not a complete proxy for malnutrition, and other factors beyond simple weight or broader nutritional status likely impact cognition. Other potentially important physiological differences may exist between these two subgroups. Restrictors tend to have long and enduring periods of starvation whereas those with binge-purge patterns often weather peaks and valleys of nutritional intake; they might not be subjected to the chronicity and intensity of deprivation experienced by AN-R patients. Regular caloric consumption that occurs during acute refeeding might constitute larger deviations from baseline for patients with AN-R compared to AN-BP, and might thereby facilitate more rapid improvement in cognitive status.

Though several prior studies have examined cognitive deficits in AN, many used adolescent populations, cross sectional designs, or different assessment methods [2, 4, 11–14, 38]. Three previous studies examined cognitive impairment in adults with AN using RBANS [3, 5, 39]. However, Keifer et al. [3] and [5] used the same patient cohort. Since Keifer et al. [3] broke the cohort into AN subtypes we will further examine their results in reference to ours in addition to evaluating those of Mikos et al. (2008) [39].

Keifer et al. found improvements in RBANS scores after treatment only in patients with AN-BP, which our opposite to our findings. However, our studies differ in several ways. Our patients were tested at significantly lower BMIs (BMI at baseline 12.7 + 1.1 and stabilization 14.6 + 1.2 compared to baseline 16.58 + 2.49 and post treatment 19.28 + 1.49 in [3]). Unexpectedly, both our AN-R and AN-BP patients had higher baseline RBANS scores than those of Keifer et al. [3]. Since the opposite would be expected given poorer nutritional status observed in our population as measured by BMI and %IBW it is difficult to account for the differences observed between our study and that of Keifer et al. [3].

Mikos et al. (2008) [39] did not show significant improvements in RBANS scores from admission to discharge though they did not break their population into subtypes and their admission BMI was much higher than our’s at 17.09. They did find significant differences in admission RBANS scores and scores at 2 year follow up. We have not followed our cohort long enough to determine if we would see similar findings. Despite lower BMI, our AN-BP patients had higher RBANS scores than the cohort of Mikos et al. (2008) [39] though our AN-R patient’s showed similar scores. These results again come as suprising given differences in BMI in our respective populations.

We found no relationship between changes in cognition and serum leptin levels, suggesting the adipokine does not mediate the measured cognitive domains assessed in this study. However, our study was not designed or powered to specifically detect this outcome and our conclusions in this regard are therefore limited.

### Limitations

Our study has several limitations. First, to measure cognition we used RBANS rather than more comprehensive and perhaps more sensitive assessments. However, our patient population would have had difficulty tolerating lengthier and comprehensive testing batteries given significant weakness and fatigue observed for BMIs averaging 12.7. The time between initial testing and repeat testing for our patients was generally between 1 and 3 weeks, significantly shorter than testing intervals in previous studies which have ranged from 1 to 12 months ([2–4, 11]). However, we were interested in the impact of acute refeeding, a shorter process than residential or outpatient treatment. The study most consistent with ours regarding timeframe is that of Keifer et al. [3], although our results differed as discussed above. Our final study population included only 39 patients as opposed to the original power calculation requiring 40 patients. However, inclusion of one additional patient is unlikely to have majorly altered our findings.

Strengths of our study include our unique patient population with regard to low weights and medical acuities. We also employed a control group which was not used by Keifer et al. [3]. Our analyses looked at differences in diagnostic subgroups and controlled for comorbid symptoms of anxiety and depression which previous studies have neglected ([2–4, 11]).

## Conclusion

In summary, our study showed no clinically meaningful decrements in cognitive functioning in a large cohort of significantly malnourished patients with anorexia nervosa. We found statistically significant increases in cognitive test scores amongst patients with AN-R relative to healthy controls after acute refeeding that were independent of improvements in comorbid psychiatric symptoms and unrelated to changes in serum leptin levels. These findings argue against theoretical concerns regarding cognitive function in severely ill patients.

## Data Availability

The data that support the findings of this study are available on request from the corresponding author. The raw data are currently stored by the authors at the NIMH Data Archive.

## Abbreviations

AN: Anorexia nervosa
AN-BP: Anorexia nervosa, binge purge subtype
AN-R: Anorexia nervosa, restrincting subtype
BAI: Beck Anxiety Inventory
BDI: Beck Depression Inventory
BMI: Body mass index
DSM: Diagnostic and Statistical Manual
IBW: Ideal body weight
OCI: Obsessive compulsive inventory
RBANS: Repeatable Battery for the Assessment of Neuropsychological Status
TMT – B/D: Trail Making Test, Part B/D
WRAT: Wide range achievement test

## References

[1] Brand-Gothelf A, Leor S, Apter A, Fennig S (2014). The impact of comorbid depressive and anxiety disorders on severity of anorexia nervosa in adolescent girls. J Nerv Ment Dis.

[2] Hatch A, Madden S, Kohn MR, Clarke S, Touyz S, Gordon E, Williams LM (2010). In first presentation adolescent anorexia nervosa, do cognitive markers of underweight status change with weight gain following a refeeding intervention?. Int J Eat Disord.

[3] Keifer E, Duff K, Beglinger LJ, Barstow E, Andersen A, Moser DJ (2010). Predictors of neuropsychological recovery in treatment for anorexia nervosa. Eat Disord.

[4] Lozano-Serra E, Andres-Perpina S, Lazaro-Garcia L, Castro-Fornieles J (2014). Adolescent anorexia nervosa: cognitive performance after weight recovery. J Psychosom Res.

[5] Moser DJ, Benjamin ML, Bayless JD, McDowell BD, Paulsen JS, Bowers WA (2003). Neuropsychological functioning pretreatment and posttreatment in an inpatient eating disorders program. Int J Eat Disord.

[6] Halmi KA, Eckert E, Marchi P, Sampugnaro V, Apple R, Cohen J (1991). Comorbidity of psychiatric diagnoses in anorexia nervosa. Arch Gen Psychiatry.

[7] Kaye WH, Bulik CM, Thornton L, Barbarich N, Masters K (2004). Comorbidity of anxiety disorders with anorexia and bulimia nervosa. Am J Psychiatry.

[8] Franko DL, Keshaviah A, Eddy KT, Krishna M, Davis MC, Keel PK, Herzog DB (2013). A longitudinal investigation of mortality in anorexia nervosa and bulimia nervosa. Am J Psychiatry.

[9] Long CG, Fitzgerald KA, Hollin CR (2012). Treatment of chronic anorexia nervosa: a 4-year follow-up of adult patients treated in an acute inpatient setting. Clin Psychol Psychother.

[10] Wentz E, Gillberg IC, Anckarsäter H, Gillberg C, Råstam M (2009). Adolescent-onset anorexia nervosa: 18-year outcome. Br J Psychiatry.

[11] Green MW, Elliman NA, Wakeling A, Rogers PJ (1996). Cognitive functioning, weight change and therapy in anorexia nervosa. J Psychiatr Res.

[12] Kingston K, Szmukler G, Andrewes D, Tress B, Desmond P (1996). Neuropsychological and structural brain changes in anorexia nervosa before and after refeeding. Psychol Med.

[13] Kjaersdam TG, Fagerlund B, Jepsen JR, Bentz M, Christiansen E, Valentin JB, Thomsen PH (2016). Are weight status and cognition associated? An examinatin of cognitive development in children and adolescents with anorexia nervosa 1 year after first hospitalization. Eur Eat Disord Rev.

[14] Nikendei C, Funiok C, Pfuller U, Zastrow A, Aschenbrenner S, Weisbrod M (2011). Memory performance in acute and weight-restored anorexia nervosa patients. Psychol Med.

[15] Steinhausen HC (2002). The outcome of anorexia nervosa in the 20th century. Am J Psychiatr.

[16] Arcelus J, Mitchell AJ, Wales J, Nielsen S (2011). Mortality rates in patients with anorexia nervosa and other eating disorders. A meta-analysis of 36 studies. Arch Gen Psychiatry.

[17] Hoang U, Goldacre M, James A (2014). Mortality following hospital discharge with a diagnosis of eating disorder: national record linkage study, England, 2001-2009. Int J Eating Disord.

[18] Huas C, Caille A, Godart N, Foulon C, Pham-Scottez A, Divac S (2011). Factors predictive of ten-year mortality in severe anorexia nervosa patients. Acta Psychiatr Scand.

[19] Ahima RS, Flier JS (2000). Leptin. Annu Rev Physiol.

[20] Eckert ED, Pomeroy C, Raymond N, Kohler PF, Thuras P, Bowers CY (1998). Leptin in anorexia nervosa. J Clin Endocrinol Metab.

[21] Haas V, Onur S, Paul T, Nutzinger DO, Bosy-Westphal A, Hauer M (2005). Leptin and body weight regulation in patients with anorexia nervosa before and during weight recovery. Am J Clin Nutr.

[22] Haluzík M, Papežová H, Nedvídková J, Kábrt J (1999). Serum leptin levels in patients with anorexia nervosa before and after partial refeeding, relationships to serum lipids and biochemical nutritional parameters. Physiol Res.

[23] Hebebrand J, Blum WF, Barth N, Coners H, Englaro P, Juul A (1997). Leptin levels in patients with anorexia nervosa are reduced in the acute stage and elevated upon short-term weight restoration. Mol Psychiatry.

[24] Monteleone P, Di Lieto A, Tortorella A, Longobardi N, Maj M (2000). Circulating leptin in patients with anorexia nervosa, bulimia nervosa or binge-eating disorder: relationship to body weight, eating patterns, psychopathology and endocrine changes. Psychiatry Res.

[25] Dagon Y, Avraham Y, Magen I, Gertler A, Ben-Hur T, Berry EM (2005). Nutritional status, cognition, and survival: a new role for leptin and AMP kinase. J Biol Chem.

[26] Dhar M, Wayman G a, Zhu M, Lambert TJ, Davare M a, Appleyard SM (2014). Leptin-induced spine formation requires TrpC channels and the CaM kinase Cascade in the Hippocampus. J Neurosci.

[27] Dhar, M., Zhu, M., Impey, S., Lambert, T. J., Bland, T., Karatsoreos, I. N., … Wayman, G. A. (2014b). Leptin induces hippocampal synaptogenesis via CREB-regulated MicroRNA-132 suppression of p250GAP. Mol Endocrinol (Baltimore, Md.), 28, 1073–87. doi:10.1210/me.2013-1332. 10.1210/me.2013-1332 PMC4075163 24877561

[28] Morrison CD. Leptin signaling in brain: a link between nutrition and cognition? Biochim Biophys Acta Mol basis Dis. 2009. 10.1016/j.bbadis.2008.12.004. 10.1016/j.bbadis.2008.12.004 PMC2670357 19130879

[29] American Psychiatric, A., & Force, D. S. M. T (2013). Diagnostic and statistical manual of mental disorders : DSM-5.

[30] Randolph C (2012). Repeatable battery for the assessment of neuropsychological status update (RBANS update).

[31] Reitan R (1955). The relation of the trail making test to organic brain damage. J Consult Psychol.

[32] DesRosiers G, Kavanagh D (1987). Cognitive assessment in closed head injury: stability, validity and parallel forms for two neuropsychological measures of recovery. Int J Clin Neuropsychol.

[33] Wilkinson GS, Robertson GJ (2006). Wide range achievement test 4: professional manual.

[34] Beck AT, Steer RA, Brown GK (1996). Beck depression inventory–second edition: manual.

[35] Beck AT, Steer RA (1990). Beck anxiety inventory: manual.

[36] Foa EB, Kozak MJ, Salkovskis PM, Coles ME, Amir N (1998). The validation of a new obsessive-compulsive disorder scale: the obsessive-compulsive inventory. Psychol Assess.

[37] Randolph C, Tierney MC, Mohr E, Chase TN (1998). The repeatable battery for the assessment of neuropsychological status (RBANS): preliminary clinical validity. J Clin Exp Neuropsychol.

[38] Harper JA, Broderick B, Van Enkevort E, McAdams CJ. Neuropsycholigcal and cognitive correlates of recovery in anorexia nervosa. Eur Eat Disorder Rev.2017;25(6):491–500. 10.1002/erv.2539 PMC5653453 28799287

[39] Mikos A, McDowell B, Moser D, Bayless J, Bowers W, ANserson A, Paulson J. Stability of neuropsychological performance in anorexia nervosa. Ann.Clin Psychiatry. 2008;20(1):9–13. 10.1080/10401230701844836 PMC3808087 18297581

